# MITF and BRN2 contribute to metastatic growth after dissemination of melanoma

**DOI:** 10.1038/s41598-017-11366-y

**Published:** 2017-09-07

**Authors:** Jacinta L. Simmons, Carly J. Pierce, Fares Al-Ejeh, Glen M. Boyle

**Affiliations:** 1 Cancer Drug Mechanisms Group, Department of Cell and Molecular Biology, QIMR Berghofer Medical Research Institute, Herston, Queensland 4006 Australia; 2 Personalised Medicine Team, Department of Cell and Molecular Biology, QIMR Berghofer Medical Research Institute, Herston, Queensland 4006 Australia

## Abstract

Melanoma tumors are highly heterogeneous, comprising of different cell types that vary in their potential for growth and invasion. Heterogeneous expression of the Microphthalmia-associated Transcription Factor (MITF) and the POU domain transcription factor BRN2 (POU3F2) has been found in malignant melanoma. Changing expression of these transcription factors as the disease progresses has been linked to the metastatic mechanism of phenotype switching. We therefore investigated the effects of MITF and BRN2 expression in melanoma growth and metastasis. Depletion of MITF resulted in a cell population that had a slowed cell cycle progression, was less invasive *in vitro* and had hindered tumor and metastasis forming ability in mouse xenograft studies. BRN2 depletion left a cell population with intact proliferation and invasion *in vitro*; however metastatic growth was significantly reduced in the mouse xenograft model. These results suggest that the proliferative population within melanoma tumors express MITF, and both MITF and BRN2 are important for metastatic growth *in vivo*. This finding highlights the importance of BRN2 and MITF expression in development of melanoma metastasis.

## Introduction

It has long been recognized that tumor cells can display remarkable variability in almost every phenotypic trait. This phenotypic heterogeneity may result from genetic or non-genetic influences, including epigenetic and micro-environmental inputs. Heterogeneity within tumors is thought to play a critical role in drug resistance and relapse following treatment^1^. It is also emerging that heterogeneous sub-populations can influence the metastatic behavior of other cell populations within the same tumor^2^. It is important to understand the biology and interactions occurring in heterogeneous tumors to enable effective targeting of treatment to prevent metastasis and recurrence.

A model for melanoma intra-tumoral heterogeneity has been proposed, where proliferation and invasion is driven by specific gene expression networks in a dynamic manner^3^. The model, known as “phenotype-switching”, predicts that all cells are capable of becoming invasive. Highly proliferative cells within the primary tumor change or “switch” phenotype possibly due to micro-environmental signals to more slowly proliferating but highly invasive cells that are capable of metastasis^4^. These invasive cells can migrate to a distant site, and either lay dormant or revert phenotype to that of the highly proliferative primary tumor and form a new metastasis. The invasive cells therefore require the ability to “switch” back to a proliferative state to enable metastatic growth. Indeed it has been shown that expression of many proteins is reversible in melanoma^5, 6^. Immunohistochemical analysis of matched primary melanoma and metastatic tissues revealed similarities between the tumors with both displaying proliferative and invasive regions supporting the model^7^.

Two key molecules identified in melanoma phenotype switching are the Microphthalmia-associated Transcription Factor (MITF) and the POU domain transcription factor POU3F2 (better known as BRN2)^4, 8^. MITF is a lineage-determining transcription factor critical for regulation of the melanocytic lineage during development and implicated as both a melanoma tumor suppressor and oncogene^9, 10^. BRN2 is a neural-lineage transcription factor involved in cell migration during neural crest development, that shows increased expression in melanoma^11, 12^. Depletion of BRN2 has been shown to reduce MITF levels^11, 13–15^ and BRN2 induces transcription of MITF. n contrast to these findings expression of BRN2 and MITF was mutually-exclusive in two distinct sub-populations within melanoma patient biopsies^8^. Importantly, BRN2 represses MITF expression by binding to its promoter preventing transcription^8^. We have additionally shown a role for MITF in reducing BRN2 protein levels by controlling expression of miR-211, highlighting a feedback loop for the levels of these transcription factors^16^. Intra-vital imaging of an engineered mouse melanoma cell line, expressing GFP driven by a BRN2 promoter, has demonstrated motile, invasive cells leaving the site of the primary tumor had high expression of BRN2 while lacking pigmentation suggesting loss of MITF expression^17^. These authors showed that although switching was possible in both directions, switching from a BRN2-high state to a BRN2-low state was favored over -low to -high switching in metastases. Many additional factors have been linked to the ‘invasive’ phenotype and phenotype switching in melanoma including TGFβ, WNT5A, AXL, TNFα/NF-κB, JUN, TEAD and various EMT regulators (as reviewed in refs 18 and 19). Interestingly, while these factors often share similar transcriptional profiles, BRN2 has not been significantly linked to these and its precise role in the invasive phenotype remains unclear.

As expression of MITF and BRN2 is mutually exclusive in patient tumors and xenografts, we investigated the role of each of these transcription factors in the phenotype of the melanoma tumor cell population. We designed a system to knockdown expression of either BRN2 or MITF by inducibly expressing a short hairpin RNA targeting each of the transcription factors. Our results show that while MITF controls proliferation, migration and invasion *in vitro*, expression of both MITF and BRN2 was important for metastatic growth *in vivo*. These results are in contrast to previous findings, and highlight the importance of expression of both BRN2 and MITF transcription factors in development of melanoma metastasis.

## Results

### Development of MITF or BRN2 knockdown cell lines using inducible shRNA targeting

**Figure 1:**
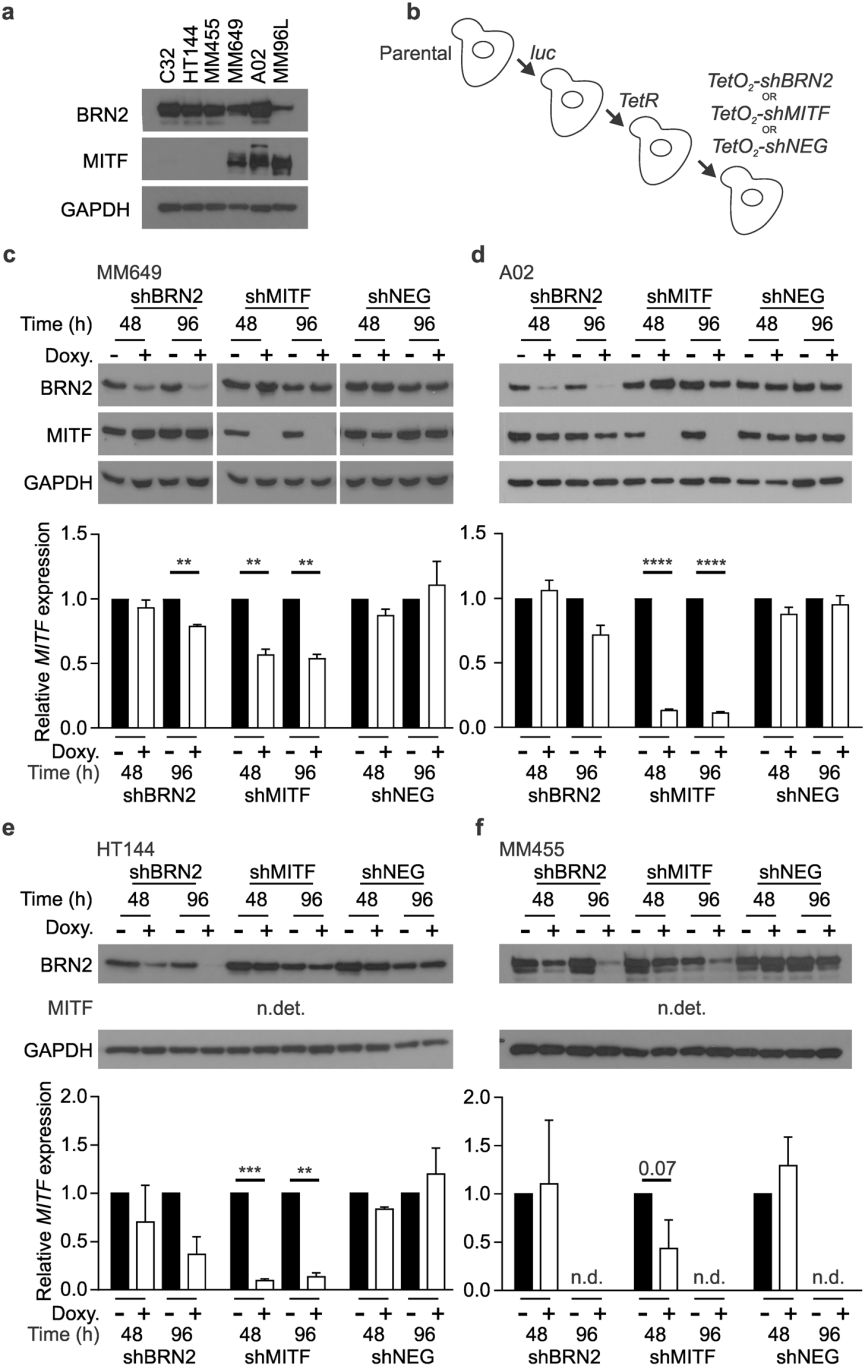
Generation of inducible MITF and BRN2 knockdown in human melanoma cell lines. (**a**) MITF and BRN2 expression determined by western blot analysis of a panel of human melanoma cell lines. (**b**) Schema of viral transduction strategy used to produce cells with doxycycline-inducible induction of shRNA targeting either MITF or BRN2 in melanoma cell lines. Western blot analysis was performed on (**c**) MM649, (**d**) A02, (**e**) HT144 and (**f**) MM455 cells 48 and 96 h after induction of shRNA expression. Lower panels show qRT-PCR detection of MITF mRNA expression after depletion of either BRN2 or MITF in all cell lines. Black blocks, vehicle only; white blocks, doxycycline; error bars, mean +/− SD. **P < 0.01, ***P < 0.001, unpaired t-test. SD, standard deviation; n.det., not detected; n.d., not determined. Full-length blots are presented in Supplementary Figures S9 and S10.

We initially identified melanoma cell lines that had expression of both BRN2 and MITF. Western blot analysis showed the expression level of both MITF and BRN2 in a panel of BRAF^V600E^ mutant metastatic melanoma cell lines (Fig. 1a). C32, HT144 and MM455 were classified as MITF^low^ (increased exposure time shown in Supplementary Figure S1a) while MM649, MM96L and A02 were MITF^high^. The expression of BRN2 varied in both groups, but trended lower in two of three MITF^high^ lines. These cell lines were sequentially transduced with lentivirus’ expressing luciferase, tetracycline (Tet) repressor and shRNA targeting either MITF, BRN2 or lacZ as a negative control (shNEG) under the control of the CMV/TetO_2_ promoter (Fig. 1b). Western blot analysis after the addition of doxycycline to cells showed depletion of MITF in MITF^high^ lines within 48 h and BRN2 within 96 h in all cell lines (Fig. 1c–f; two MITF^high^ - MM649 and A02, two MITF^low^ - HT144 and MM455). MITF mRNA levels in both MITF^low^ (HT144 and MM455) and MITF^high^ (MM649 and A02) cell lines were determined by qRT-PCR, due to difficulty in detection by western blot analysis in MITF^low^ cells. Analysis similarly showed depletion of MITF mRNA within 48 h of doxycycline addition in all lines (Fig. 1e,f) although not statistically significant in MM455. However, a known MITF target SNAIL^20^, was also down-regulated in MM455 cells following MITF depletion (Supplementary Figure S1b). Interestingly, BRN2 depletion reduced levels of MITF mRNA at 96 h post-doxycycline treatment in 3 of the 4 cell lines (data unreliable for MM455 due to very low levels) although the MITF protein level was less variable.

### Expression profiling reveals complementary transcriptional patterns following BRN2 or MITF knock-down

**Figure 2:**
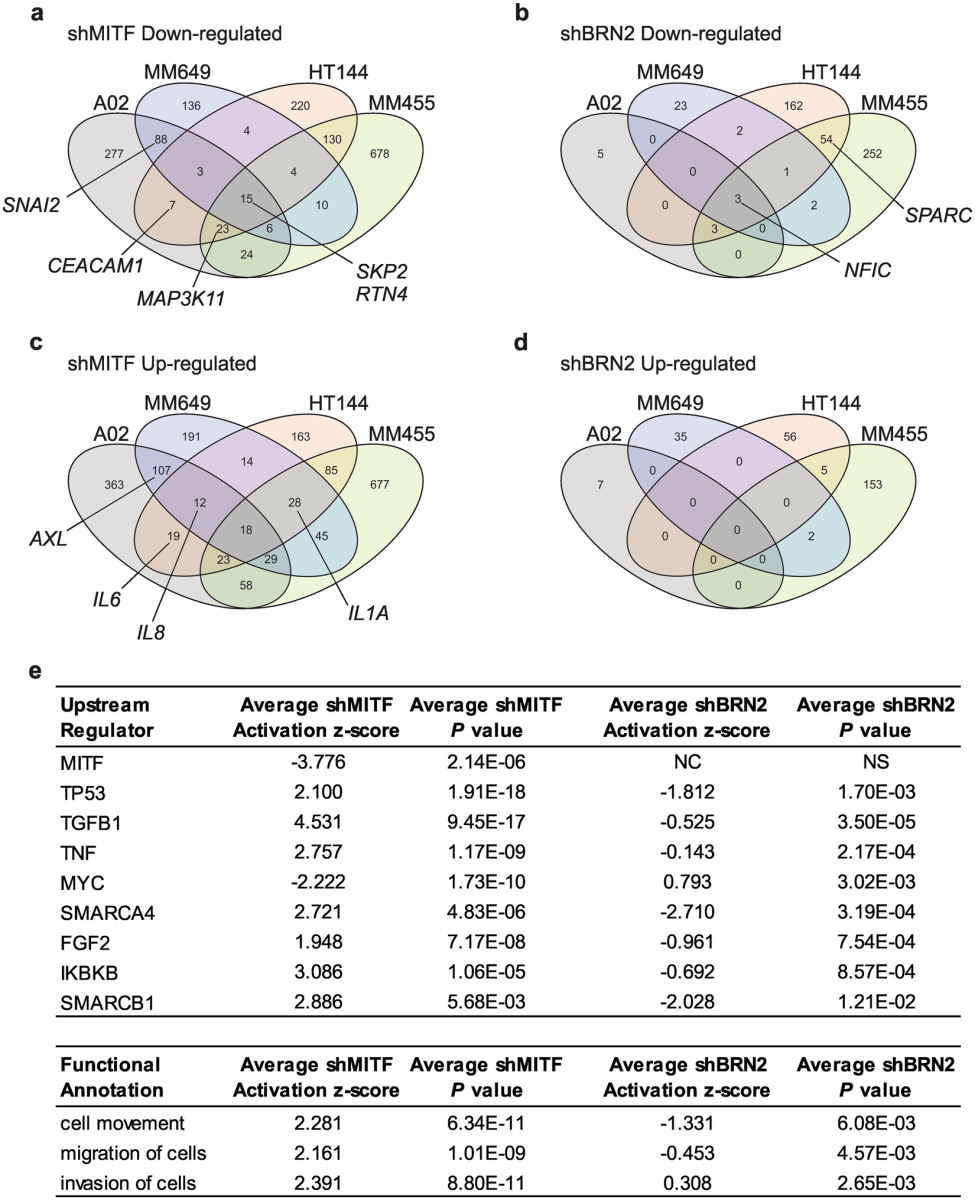
Expression profiling of cells with MITF or BRN2 knockdown. Entities at least 1.5 fold down- or up-regulated following induction of either shMITF [(**a**,**c**)] or shBRN2 [(**b**,**d**)] were identified by firstly comparing to the same uninduced cells, then by comparing to shNEG versus uninduced cells to account for changes potentially caused by shRNA expression or doxycycline exposure. Full data can be found in Supplementary Tables S1–S2. Selected validated targets are indicated (Supplementary Figure S2). (**e**) Selected Upstream regulator or Functional annotation pathways showing average z-score and *P* value for all 4 cell lines expressing shMITF or shBRN2. Full data is contained in Supplementary Tables S3–S8. NC, not called; NS, not significant.

We used gene expression profiling to understand the contribution of BRN2 and MITF in the cellular population. Total RNA was extracted from cells expressing shBRN2, shMITF or shNEG, treated with doxycycline or vehicle only as a control for 96 hours. Known targets of MITF and BRN2 transcriptional control were differentially expressed in individual cell lines (Supplementary Tables S1–S2). There was generally a low level of overlapping targets between each line, although there were more genes differentially expressed when comparing the MITF^low^ or MITF^high^ cells (Fig. 2a–d). Additionally, BRN2 depletion in MITF^low^ cells resulted in significant changes in gene expression whereas BRN2 depletion in MITF^high^ cells altered expression of fewer genes. Differential expression of selected molecules in melanoma cell lines following knockdown of MITF or BRN2 was confirmed using qRT-PCR (Supplementary Figure S2a,b). Global pathway analysis showed significantly similar transcriptional alterations following MITF knockdown in all melanoma cell lines as indicated by either the predicted upstream molecules or functional pathway annotation that would elicit the observed gene expression changes (Supplementary Tables S3–S4). Interestingly, similar upstream regulators and pathways were significantly altered in melanoma cell lines following BRN2 knockdown, although not MITF and generally with an opposite effect (indicated by the activation z-score) to the changes found with MITF depletion (Fig. 2e; Supplementary Tables S5–S8). This data suggests that MITF and BRN2 may determine opposing and complementary transcriptional programs in melanoma tumor cell populations.

### MITF expression is required to maintain cell proliferation *in vitro*

**Figure 3:**
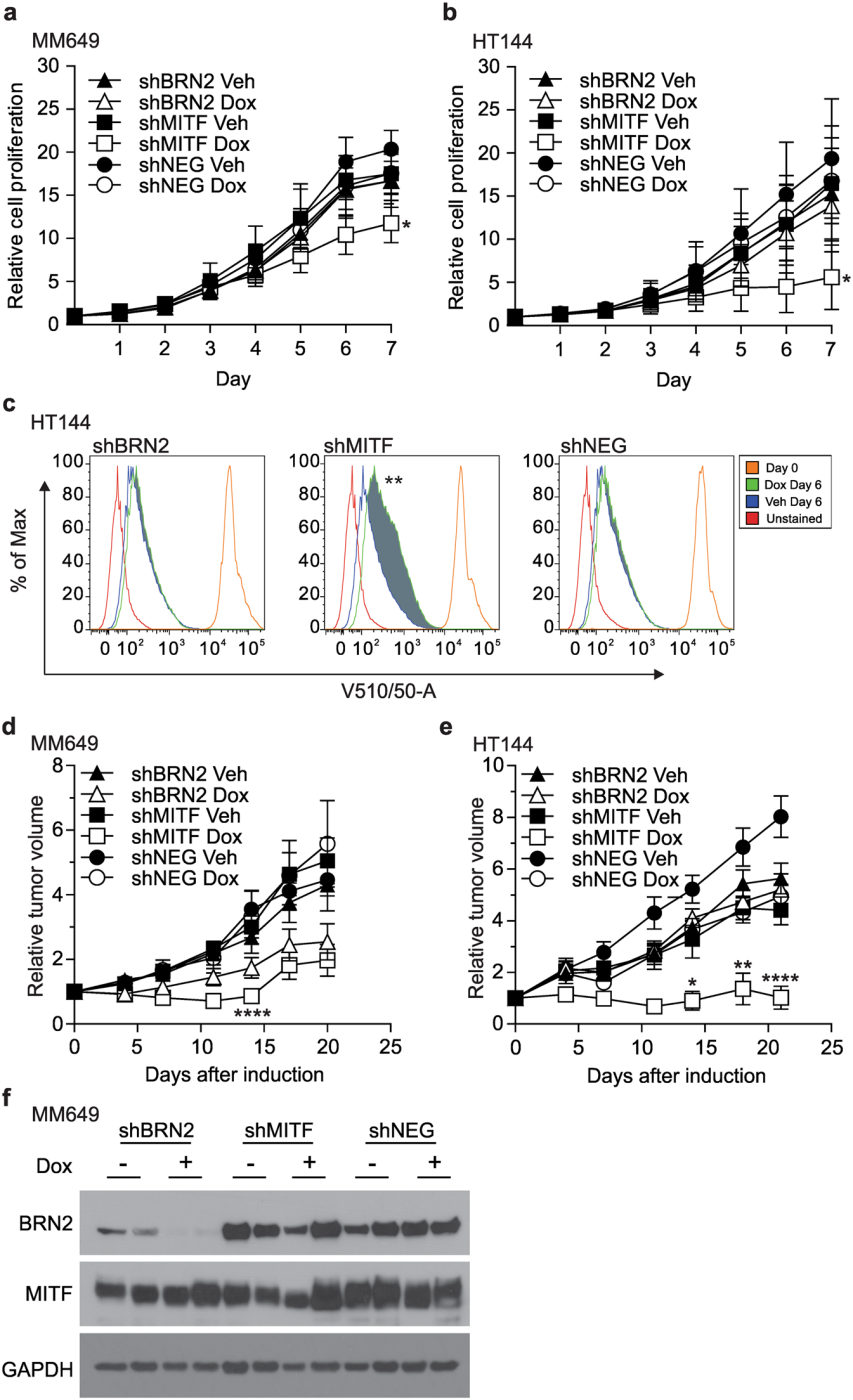
Impact of MITF or BRN2 knockdown on cellular proliferation *in vitro* or *in vivo*. (**a**) MM649 or (**b**) HT144 cells were seeded into 96-well plates, induced with doxycycline for 7 days and a Sulforhodamine B assay was used to measure cellular proliferation rate. Values indicate mean +/− SD. *P < 0.05, unpaired t-test. (**c**) Changes in the cell cycle rate were measured by incubation of Cell Trace Violet stained cells for 6 days in the presence of doxycycline and measuring the change in staining by FACS. SED% positive was used to quantitatively measure changes in cell staining. **P < 0.01, unpaired t-test. (**d**) MM649 or (**e**) HT144 cells with inducible depletion of MITF or BRN2 were injected sub-cutaneously into the right and left flanks of 5-week-old male BALB/c *Foxn1* ^*nu*^ (nude) mice, one shRNA per mouse. Expression of shRNA in established tumors was induced by the addition of doxycycline to drinking water when tumors reached approximately 50 mm^3^ (nominated Day 0). Values indicate mean +/− SEM, n = 5 mice per group, 10 tumors in total for MM649; n = 6 mice per group, 12 tumors in total for HT144. *P < 0.05, ****P < 0.0001, Mann-Whitney test. **(f)** Western blot analysis of MITF and BRN2 expression in MM649 tumors on completion of the experiment (Day 20 post-doxycycline treatment). Black shapes, vehicle only; white shapes, doxycycline. SD, standard deviation; SEM, standard error of the mean. Full-length blots are presented in Supplementary Figure S11.

We measured the effects of transcription factor expression on proliferation for 7 days after inducible knock-down of BRN2 or MITF. There was a significant reduction in cellular proliferation when cells were depleted of MITF and only expressing BRN2 in both the MITF^high^ (MM649, Fig. 3a, *P* = 0.0359; A02, Supplementary Figure S3a, *P* = 0.0065; unpaired t-test) and MITF^low^ cell lines (HT144, Fig. 3b, *P* = 0.0294; MM455, Supplementary Figure S3b, *P* = 0.0046; unpaired t-test) at day 7 after doxycycline addition compared to vehicle only controls. Knock-down of MITF level using siRNA in MM649 and HT144 (Supplementary Figure S3c) additionally resulted in reduced proliferation at day 3 and 5 following transient transfection (MM649, Supplementary Figure S3d, *P* < 0.0001; unpaired t-test; HT144, Supplementary Figure S3e *P* < 0.0001; unpaired t-test). There was no significant difference in proliferation when cells were depleted of BRN2 by inducible shRNA compared to the cell population expressing both factors (shNEG; Fig. 3a,b; Supplementary Figure S3a,b). While BRN2 knockdown cells were able to efficiently form colonies to the same level as the cellular population expressing control shNEG, this ability was severely hindered by the depletion of MITF (data not shown). The cell population lacking MITF were viable, as removal of doxycycline after 7 days in colony formation assays resulted in re-growth of colonies in similar numbers to those not exposed to doxycycline (data not shown).

Reduction of MITF expression has been previously associated with G1 arrest^14, 21^, senescence^22^ or increased apoptosis^23^. However, cell cycle analysis showed no uniform changes in the proportion of G1 or apoptotic cells in cells depleted of MITF or BRN2 (Supplementary Figure S4a,b). Similarly, western blot analysis revealed no PARP-1 cleavage in cells depleted of MITF or BRN2 (Supplementary Figure S5). Furthermore, the number of senescent cells remained unchanged following inducible depletion of either MITF or BRN2 in these lines (data not shown). Using retention of a membrane incorporated dye, we found the reduced proliferation observed in MITF-depleted HT144 cells was due to a slow cycling phenotype, that is, the cell cycle in these cells progresses at a slower rate (Fig. 3c, *P* = 0.0023; unpaired t-test). No differences in dye retention were observed in the cells expressing shRNA targeting BRN2 or in the shNEG control population. This data indicates that MITF is crucial in maintaining proliferation, due to the cell population cycling more slowly upon depleted of MITF.

### MITF expression in the population is required for primary tumor growth while BRN2 is dispensable

We assessed if MITF and BRN2 expression is required for primary melanoma tumor growth using mouse xenograft studies. MM649 (MITF^high^) or HT144 (MITF^low^) cells injected intra-dermally on the flank of BALB/c *Foxn1* ^*nu*^ (nude) mice were allowed to form tumors (approximately 50 mm^3^) before depletion of MITF or BRN2 by induction of shRNA expression with doxycycline. Depletion of MITF or BRN2 from established tumors of MM649 cells (MITF^high^) resulted in an initial reduction in tumor volume of both shBRN2 and shMITF expressing tumors (Fig. 3d). After 7 days of doxycycline treatment, BRN2 knockdown (shBRN2) tumors recommenced growth; however tumors ablated of MITF (shMITF) continued to decrease in volume resulting in a significantly reduced tumor volume until day 14 after initiation of doxycycline treatment (Fig. 3d, Day 14, *P* < 0.0001; Mann-Whitney test; raw individual tumor data shown in Supplementary Figure S6a). MM649 tumor volume after BRN2 knockdown was not significantly different to the vehicle only control (Fig. 3d, Day 14, *P* = 0.1777, Mann-Whitney test). HT144 cells, with a considerably lower level of MITF protein, form slower growing tumors compared to MM649 when xenografted into BALB/c *Foxn1* ^*nu*^ mice (time to approximately 50 mm^3^ tumor volume; HT144 – 14 days, MM649 – 7 days; data not shown). When MITF was further depleted in HT144 cells leaving BRN2 expressed in the population, tumor growth was again significantly reduced (Fig. 3e, Day 14, *P* = 0.0244; Mann-Whitney test; raw individual tumor data shown in Supplementary Figure S6b). However, it was apparent that the MM649 tumors expressing shRNA to target MITF began to regrow after 14 days exposure to doxycycline. Immunohistochemistry was used to monitor MITF and BRN2 expression in excised MM649 and HT144 tumors selected at random from the cohort after 14 days doxycycline treatment. The staining revealed reduced expression of BRN2 in both tumor types with shRNA targeting BRN2 (Supplementary Figure S7a,b). While knockdown of MITF was maintained in HT144 tumors expressing shMITF, analysis showed expression of MITF in regions of the MM649 tumors expressing shMITF (positive region shown in Supplementary Figure S7a). Western blot analysis of excised MM649 tumors on completion of the experiment (Day 20 post-doxycycline administration) showed BRN2 to be barely detectable in the tumors expressing shRNA targeting BRN2 while MITF was highly expressed in the shMITF expressing tumors, indicating escape from shRNA control (Fig. 3f). Taken together, these results show that MITF plays a crucial role in primary tumor growth.

### Depletion of MITF from the population, but not BRN2, reduces migration and invasion *in vitro*

**Figure 4:**
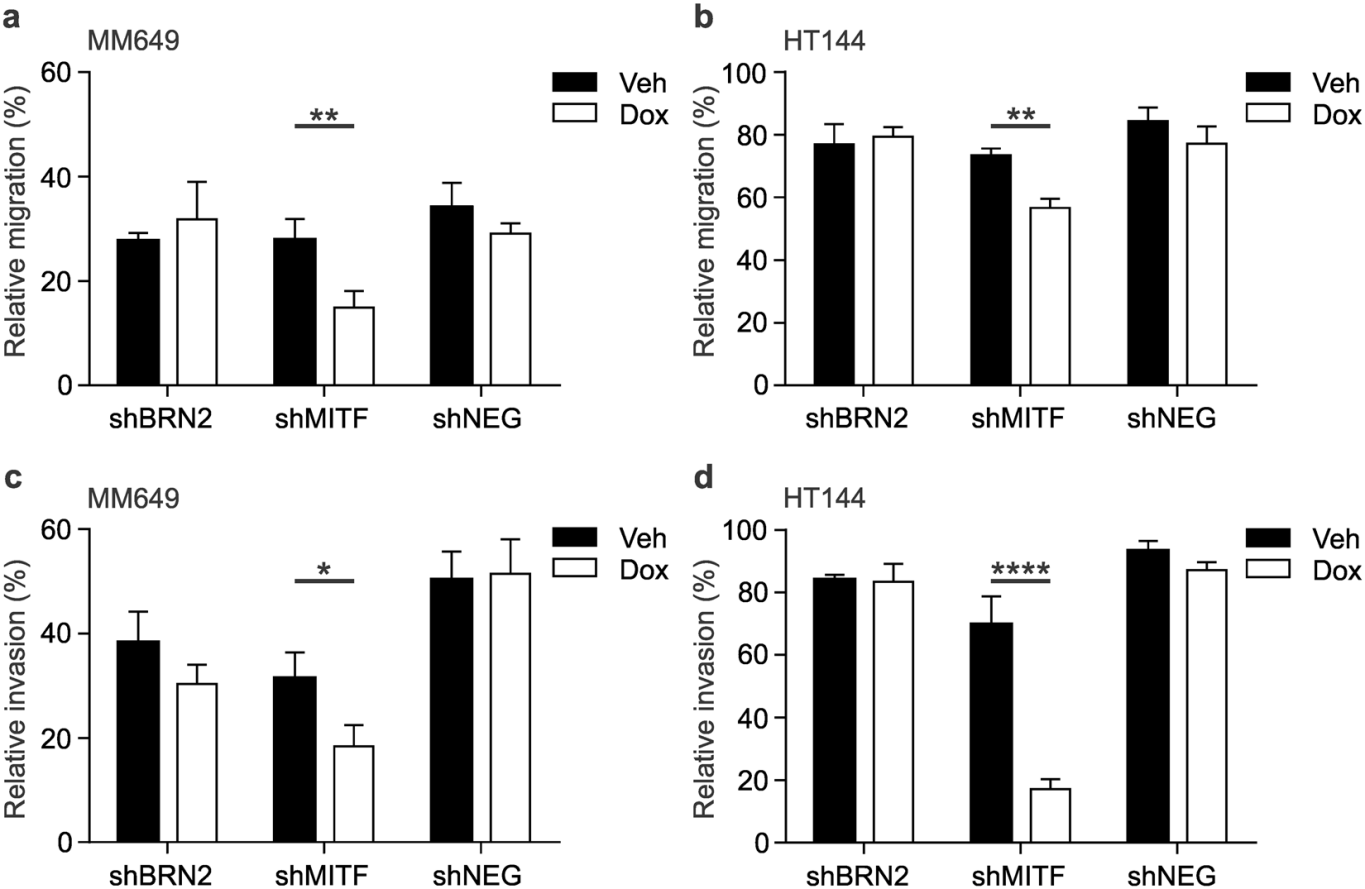
Depletion of MITF but not BRN2 reduces migration and invasion *in vitro*. (**a**,**b**) Migration or (**c**,**d**) invasion of cells depleted of MITF or BRN2. Cell migration was measured by wound healing assay, reduced migration was observed in (**a**) MM649 and (**b**) HT144 cells expressing shMITF. Quantitative cell invasion through Basement Membrane Matrix was measured in (**c**) MM649 and (**d**) HT144 cells ablated for MITF expression. Black blocks, vehicle only; white blocks, doxycycline. Values indicate mean +/− SD. *P < 0.05, **P < 0.01, ****P < 0.0001, unpaired t-test. SD, standard deviation.

The ability of cells to migrate across the cleared surface in a wound healing assay was used to measure the effect of MITF and BRN2 expression on cell migration and invasion. No measurable changes in cell movement were detected as a result of BRN2 knockdown, leaving only MITF expressed (Fig. 4a,b). However, depletion of MITF from the population significantly reduced the migration of cells (Fig. 4a,b; MM649, *P* = 0.0078; HT144, *P* = 0.0015; unpaired t-test). Similarly, invasion of cells through a basement membrane matrix was significantly reduced by depletion of MITF but not BRN2 from the population (Fig. 4c,d; MM649, *P* = 0.0293; HT144, *P* < 0.0001, unpaired t-test). This difference was not due to any growth effect, as similar results were obtained in the presence of mitomycin c (data not shown). These results suggest BRN2 expression is not required for melanoma cell migration and invasion in these cells *in vitro*, and indicate expression of MITF plays an important role in these processes.

### BRN2 and MITF are both required for growth after metastatic dissemination

**Figure 5:**
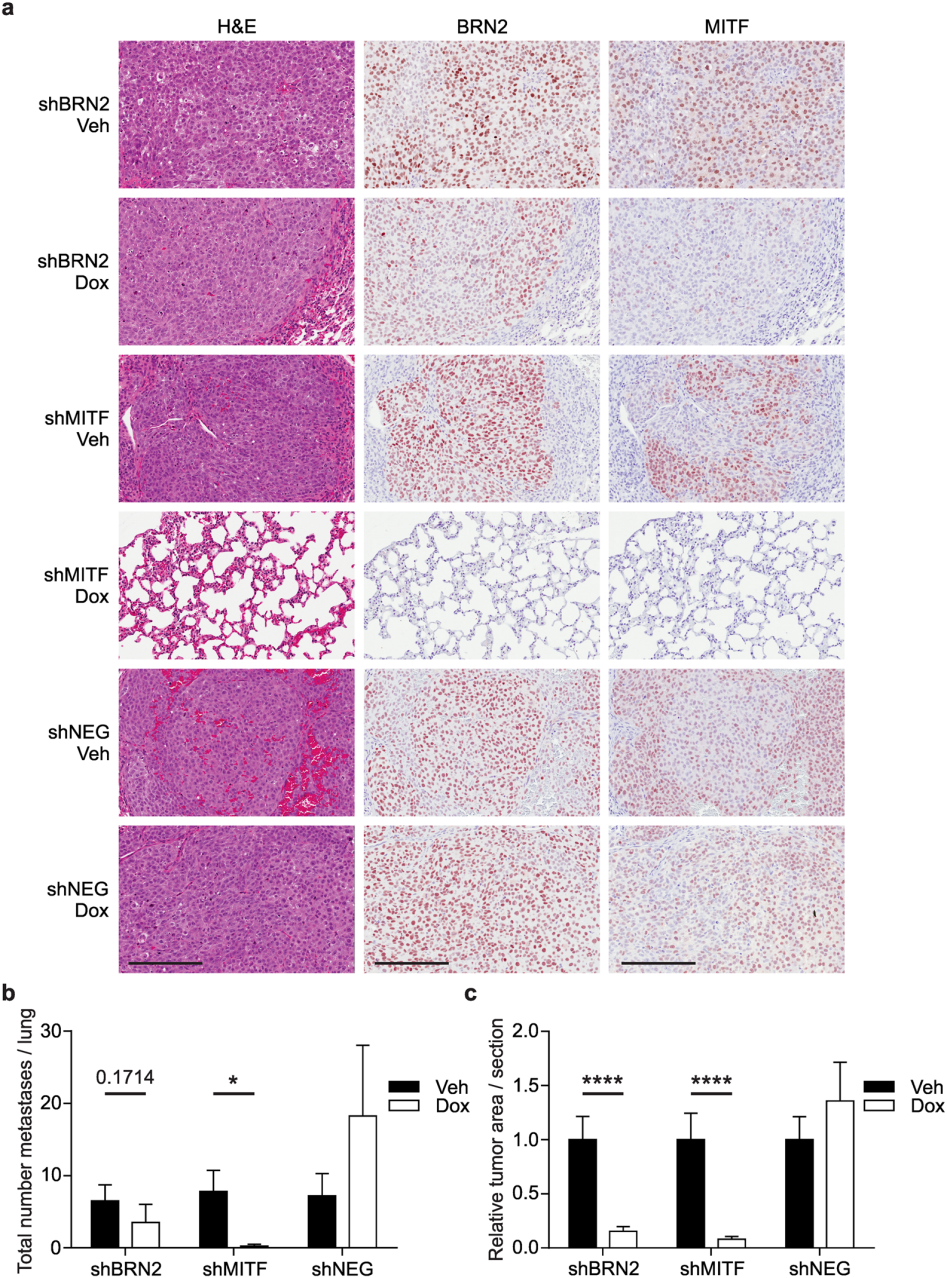
BRN2 and MITF expression is required for growth after metastatic dissemination. HT144 cells with either MITF or BRN2 shRNA were injected into the lateral tail vein of five-week-old male BALB/c *Foxn1* ^*nu*^ (nude) mice. Doxycycline administration commenced 48 h prior to injection for both cells and mice and continued for 4 weeks before switching mice back to normal drinking water. Mice were sacrificed after an additional 8 weeks or when ethically required and lungs and visible metastases removed for further analysis. (**a**) Histology and immunohistochemistry of HT144 tumors in mouse lungs. Left panels show haematoxylin and eosin staining of a lung containing melanoma tumor cells. Middle and right panels show BRN2 or MITF expression detection respectively in lungs and suspected metastases. The staining confirmed the tumor cells originated from the melanoma cell line. Scale bars, 200 µm. (**b**) Average number of HT144 metastasis found on complete sectioning of the lungs following ablation of BRN2 or MITF compared to a population that maintained expression of both BRN2 and MITF (shNEG). (**c**) Relative HT144 tumor area per lung section was calculated after complete sectioning using Genie software analysis. Data shows a significantly decreased tumor burden (both area and percentage tumor – not shown) in mice injected with cells with reduced MITF and BRN2. Black bars, vehicle only; white bars, doxycycline. Values indicate mean +/− SEM, n = 5 mice per group. *P < 0.05, ****P < 0.0001, Mann-Whitney test. SEM, standard error of the mean.

We then used an experimental metastasis xenograft model to assess the impact of BRN2 and MITF expression on the formation of lung metastasis, given the *in vitro* invasion results. As the MITF^high^ MM649 cells do not readily form lung metastases in experimental models (unpublished data), MITF^low^ HT144 cells were used for this model. Cells were injected into the lateral tail vein of five week old nude mice following 2 days treatment of cells and mice with doxycycline, and bioluminescent imaging of mice immediately following cell injection confirmed injection efficiency (data not shown). Doxycycline was withdrawn after four weeks, to enable re-expression of MITF and BRN2 to allow cell proliferation and enable growth of metastases. On completion of the experiment, formalin-fixed, paraffin-embedded lungs were completely serial sectioned and stained using haematoxylin & eosin, anti-BRN2 and anti-MITF antibodies (Fig. 5a). A significant reduction in the total number of metastases per mouse was observed when MITF was depleted for the initial 4 week period (shMITF) (*P* = 0.0238, Mann-Whitney test), while there was a non-significant decrease where the population was depleted of BRN2 (*P* = 0.1714, Mann-Whitney test) (Fig. 5b). Quantitation of metastatic growth over all serial sections of the lungs showed a significantly decreased tumor burden in both area (Fig. 5c; raw tumor area data shown in Supplementary Figure S8a) and percentage tumor (Supplementary Figure S8b) in mice injected with a population with either reduced MITF or BRN2 (*P* < 0.0001, Mann-Whitney test). These results indicate a requirement for expression of both BRN2 and MITF in the melanoma cell population for growth following metastatic dissemination.

## Discussion

Melanoma tumors are comprised of differing cell populations. This heterogeneity is thought to be due in part to a dynamic process, described as the “phenotype-switching” model, where all cells are capable of becoming invasive after switching from a proliferative phenotype. Previous studies identified two important signatures associated with phenotypic plasticity in melanoma. One signature was associated with weakly invasive but highly proliferative cells, and the other associated with slow growing cells that were highly invasive^4, 24, 25^. The proliferative signature was associated with high expression of MITF, while the invasive melanoma cells exhibited little or no expression of MITF. Lack of MITF expression presumably results in high BRN2 levels, given that MITF represses BRN2 via miR-211 expression^16^, and BRN2 binds the MITF promoter to directly repress its expression *in vitro* ^8^. This model of heterogeneity is supported by findings from patient tumors where BRN2 and MITF expression is mutually-exclusive^8^.

We investigated the contribution of the different populations found within heterogeneous tumors by inducibly knocking down expression of either BRN2 or MITF. Global analysis of differentially expressed genes showed significantly similar pathway alterations following MITF knockdown in all melanoma lines, although the level of overlap in individual genes between MITF^high^ and MITF^low^ lines was low. Additionally, depletion of BRN2 in MITF^low^ cells altered the expression of numerous genes; in contrast BRN2 depletion in MITF^high^ cells resulted in fewer expression changes. Although the exact mechanism behind this is still unclear, it is possible that differences in levels of MITF impacts the response to BRN2 depletion. However, that similar pathways or upstream factors were significantly altered in the opposite direction following BRN2 knockdown compared to the changes found with MITF knockdown is of interest. These changes would not appear to be as a result of the described reciprocal regulation of MITF and BRN2, as only early transient changes in level were observed with knockdown of MITF or BRN2 (Fig. 1c–f). This finding potentially supports those of Thurber *et al*. where members of the NOTCH pathway decreased or increased expression following BRN2 or MITF depletion respectively^15^. This data may suggest that MITF and BRN2 determine opposing transcriptional programs in cells within melanoma tumors, and interactions between the cells within a population could be important. The specific interactions between these cell types should be investigated more closely in the future.

Inducible expression of shRNA-targeting MITF in MITF^high^ or MITF^low^ cell lines reduced cellular proliferation consistent with previous reports that MITF-positive cells are proliferative. However, contrary to previous work, reduction of MITF-positive cells in the population did not induce cell cycle arrest, senescence or apoptosis^21, 23^. Instead, MITF-depleted, BRN2-positive cells showed a slow-cycling phenotype. Some of the validated expression differences identified impact cellular proliferation; reduced expression of *CEACAM1, SKP2* and *RTN4* may contribute to slowed cell cycle expression in cells with MITF knockdown^26–29^. This phenotype is significant given that MITF^low^ cells, therefore potentially BRN2-positive cells, have been reported in tumors of relapsed melanoma patients and primary tumors of poor responders to targeted (MAPKi) therapy^30^. Slow-cycling cells have been shown to contribute to tumor maintenance in the long term and to resistance to treatment^6, 31^. Therefore MITF^low^, BRN2-positive cells within the tumor environment exhibiting a slow-cycling phenotype may contribute to therapy resistant disease.

In the phenotype switching model, cells that express BRN2 are the invasive and motile cells within the primary tumor while MITF-positive cells are proliferative. As MITF is known to impact BRN2 levels, it would be expected that proliferative MITF^high^ cells be non-invasive. Interestingly our results show depletion of BRN2 from the population did not reduce invasion *in vitro* while MITF expression was found to be necessary for invasion. These results would appear to be in contrast to some previous findings. Over-expression of MITF ablated invasion *in vitro* while siRNA-mediated MITF depletion enhanced cell invasion^21, 32^. Further, over-expression of BRN2 has been shown to induce proliferation and invasion in the melanocytic lineage^33–35^, while knockdown reduces growth and migration^8, 17^. However, it should also be noted that there are different responses as a result of modulation of BRN2 or MITF, depending on the cell lines or the experimental system used. Ectopic expression of MITF has been reported to increase invasive capacity^36^. In addition, expression of miR-211, a target of MITF, either increased or decreased BRN2 levels and invasive capacity depending on the cell line and constitutive level of MITF present^16^. Nonetheless, the current study clearly shows that both transcription factors are necessary for maximal metastatic growth *in vivo*. Depletion of MITF from the population caused a decrease in both number and size of lung metastases while BRN2 depletion similarly resulted in a reduction in tumor burden overall but did not significantly reduce the total number of metastases.

The main function of BRN2 appears to be during metastatic dissemination in melanoma. Pinner *et al*. showed that migratory cells within the primary tumor as well as disseminated cells isolated from blood are BRN2^high^ cells^17^. It was similarly shown that BRN2^low^ cells are neither tumorigenic nor metastatic in mouse xenograft models indicating a requirement for BRN2 expression during metastatic spread^11^. Our model of experimental metastasis showed a reduction in metastatic growth as a result of BRN2 depletion. This data indicates that while BRN2 may be dispensable for *in vitro* models of metastatic growth it is absolutely required *in vivo*. More specifically, our results show the presence of BRN2-positive cells within the population is important for metastatic growth *in vivo*. These results are in contrast to previous findings, where the change in phenotype from a BRN2-high state to a BRN2-low state was favored over -low to -high switching in metastases^17^. Our data highlights the importance of both MITF- and BRN2-expressing cells within the population in the growth and development of melanoma metastasis.

## Materials and Methods

### Melanoma cells and culture

The melanoma cell lines and growth have been described previously^37–39^. Routine mycoplasma tests were performed using PCR and were always negative. Cell line identity was routinely checked by Short Tandom Repeat (STR) profiling with the GenePrint® 10 System (Promega, Madison, WI) according to the manufacturer’s instructions.

### Production of shRNA expressing cell lines

The BLOCK-iT Lentiviral Expression System (Life Technologies) was used to produce cell lines stably expressing doxycycline-inducible shRNA targeting, BRN2, MITF or lacZ (negative control). The shRNA sequences cloned into the pLENTI4/TO/V5-DEST vector were as follows: BRN2 shRNA, Top 5′-GCTGTTGCGCTGCGATCTTGTCTATGTTTT GGCCACTGACTGACATAGACAATCGCAGCGCAA-3′; Bottom 5′-CCTGTTGCGCTG CGATTGTCTATGTCAGTCAGTGGCCAAAACATAGACAAGATCGCAGCGCAAC-3′; MITF shRNA, Top 5′-TGCTGTACAGGTTAGGTCTGCATGATG TTTTGGCCACTGACTGACATCATGCACCTAACCTGTA-3′; Bottom, 5′-CCTGTACAGGTTAGGTGCATGATGTCAGTCAGTGGCCAAAACATCATGCAGACCTAACCTGTAC-3′. Target cell pools were transduced with lentivirus containing pLENTI6/luciferase and selected with puromycin. Pools of selected cells were then transduced with lentivirus containing pLENTI6/TR (tetracycline repressor) and selected with puromycin and blasticidin. Cells were further transduced with pLENTI4/TO/EmGFP/shRNA targeting lacZ, BRN2 or MITF, and selected with zeocin, puromycin and blasticidin. Selection antibiotic concentration was determined for each cell line by kill curve.

### Western blot analysis

Western blot analysis has been described previously^40^. Antibodies used in this study were: anti-MITF (D5G7V; Cell Signaling Technologies [CST], Danvers, MA), anti-BRN2 antibody (12137 S; CST), anti-Snail antibody (C15D5; CST), anti-GAPDH (R&D Systems, Minneapolis, MN).

### Proliferation and colony formation

To measure cell proliferation 2.5–7.5 × 10^4^ cells were seeded per well into 96-well plates and allowed to attach for 24 h before induction of shRNA expression by the addition of 50 ng/ml doxycycline into appropriate wells. Proliferation was determined by sulforhodamine B (SRB) assay as previously described^40^. For colony formation, 2.5 × 10^4^ cells were seeded per well into 6-well plates and induced with doxycycline as above 24 h after seeding, media was replaced every 3–4 days for 14 days. Colonies were fixed with methylated spirits and stained with 0.1% crystal violet in methanol.

### Migration and invasion assays

Cells were seeded into a 96-well ImageLock^TM^ plate (Essen Bioscience, Ann Arbor, Michigan) then appropriate wells induced by the addition of 50 ng/ml doxycycline to culture media after 24 h. After a further 48 h the Essen Bioscience WoundMaker^TM^ was used to wound the confluent monolayer of cells, and media replaced after washing. Cellular density in the wound was measured with two hourly images collected with the IncuCyte Zoom (Essen Bioscience). To measure invasion cells were grown to confluence in a 96-well plate coated with 1 mg/ml Basement Membrane Matrix (BMM; Corning Inc, Corning, NY). Cells were induced with doxycycline as above and a wound created as above. After wounding, 50 µl of 3 mg/ml BMM in culture media was plated over the cells. The BMM was gelled at 37 °C for 1 h then covered with culture media +/− doxycycline. Images were collected every 2 h for 96 h to measure wound cell density.

### Cell cycle analysis

2.0 × 10^6^ cells were stained with CellTrace^TM^ Violet (Life Technologies) following the manufactures protocol and cultured for 24 h. Cells were split into 3 groups, one ethanol fixed and the remaining two groups plated into fresh media, one group containing 50 ng/ml doxycycline. After 6 days cells were detached with trypsin and fixed in ice cold 70% ethanol. Fixed cells were counterstained with 5 mg/ml Propidium Iodide (PI, Sigma, St. Louis, MO) before being analyzed on the FACS Canto II (BD Biosciences, Franklin Lakes, NJ) for staining of both CellTrace^TM^ Violet and PI. FlowJo Version 9 (Tree Star Inc, Ashland, OR) was used to analyze cell trace staining and determine the percentage positivity. Cell cycle analysis was completed using ModFit LT (Version 4.0, Verity Software House, Topsham, ME).

### Xenograft studies

All animal studies were approved by the QIMR Berghofer Medical Research Institute Animal Ethics Committee (P949) and performed in accordance with the guidelines. For tumorigenicity studies 2.0 × 10^6^ cells in 50 µl of RPMI-1640/10% FCS were injected intra-dermally into each of two sites of six five-week old male BALB/c nude mice. Tumors were measured twice weekly and volume calculated in mm^3^. For experimental metastasis studies, 0.5 × 10^6^ cells in single cell suspension in 100 µl PBS were injected into the lateral tail vein of five-week old male BALB/c nude mice. Mice were fed 2 mg/ml doxycycline in 4% sucrose to induce the shRNA expression in the appropriate groups, or 4% sucrose as a control. Bioluminesent imaging of metastatic growth was performed following cell injection using 125 mg/kg luciferin (Gold Biotechnology, St Louis, MO) and the PhotonImager Optima (BioSpace Lab, France).

### Histology

Antigen retrieval for staining was for 20 min at 100 °C in a 1 mM Tris, 1 mM EDTA, pH 9 buffer. BRN2 (12137 S; CST) or MITF (D5G7V; CST) antibodies were diluted 1:100 in Da Vinci Green (Biocare Medical, Concord, CA) and incubated overnight at room temperature. Sections were then incubated in MACH 1 Universal HRP polymer (Biocare Medical) for 30 min, counterstained with Nova Red (DakoCytomation, Glostrup, Denmark) followed by haematoxylin. Additional sections were also stained with haematoxylin and eosin following standard procedures. All stained sections were scanned with the Aperio AT Turbo (Leica Biosystems, Nussloch, Germany) and images extracted in ImageScope v11 (Leica Biosystems). Total tumor area per lung was determined by staining fully serial sectioning formalin-fixed, paraffin-embedded lungs by IHC with a BRN2 antibody. After scanning with the Aperio AT Turbo the Genie macro for ImageScope (Leica Biosystems) was used to measure the total tumor area and total lung area per section.

### Tyramide Signal Amplification

De-waxed paraffin-embedded tissue sections or paraformaldehyde fixed cultured melanoma cells were microwaved in DAKO pH 6 antigen retrieval buffer for 15 min and cooled (in buffer) on ice for 30 min. Antibodies were diluted at 1:1,000 (MITF) or 1:2,000 (BRN2) in TBS and slides were incubated at room temperature for 30 min. MACH1 Universal HRP polymer (Biocare Medical) was applied to slides for 10 min then either TSA Cy3 or Fluorescein (Perkin Elmer, Waltham, MA) for a further 10 min. Each antibody was applied sequentially with microwaving after each fluorochrome. Slides were counterstained with DAPI. Images were obtained using the Nuance Multispectral imaging system (Perkin Elmer) and spectral unmixing was completed with inForm software (Perkin Elmer).

### Expression profiling and analysis

Whole genome expression profiling was as previously described^41, 42^. Entities at least 1.5 fold up- or down-regulated following induction of either shBRN2 or shMITF were identified by comparing to the same, uninduced cells, then by comparing to shNEG versus uninduced cells to account for changes caused by shRNA expression or doxycycline exposure. Significant pathways within the data were assessed using Ingenuity Pathway Analysis (IPA^®^, Qiagen) software. The data discussed in this publication have been deposited in NCBI’s Gene Expression Omnibus, and are accessible through GEO Series accession number GSE101434.

### qRT-PCR

2 µg of total RNA was reverse transcribed with SuperScript III reverse transcriptase (Invitrogen, Carlsbad, CA) following the manufacturer’s protocol. qRT-PCR was performed using the CFX384 Real-Time System (BioRad Laboratories, Hercules, CA) with SYBR Green PCR master mix (Applied Biosystems, Foster City, CA). Primers used in this study are listed in Supplementary Table S9.

### Statistical analysis

Data is expressed as mean +/− error (SD or SEM as indicated). All statistical significance was determined by GraphPad Prism (v6, La Jolla, CA) using unpaired t-tests or Mann-Whitney tests as indicated and *P* < 0.05 was considered statistically significant.

### Data availability

The data discussed in this publication have been deposited in NCBI’s Gene Expression Omnibus, and are accessible through GEO Series accession number GSE101434.

## Electronic supplementary material

Supplementary Information

Supplementary Table S1

Supplementary Table S2

Supplementary Table S3

Supplementary Table S4

Supplementary Table S5

Supplementary Table S6

Supplementary Table S7

Supplementary Table S8
