# Supplementary material for: MITF and BRN2 contribute to metastatic growth after dissemination of melanoma
Source: Sci Rep. 2017 Sep 7;7:10909. doi: 10.1038/s41598-017-11366-y (PMC5589904; doi:10.1038/s41598-017-11366-y)
Supplement: Supplementary file 1 — Supplementary Information [file 41598_2017_11366_MOESM1_ESM.pdf]

**MITF and BRN2 contribute to metastatic growth after dissemination of melanoma.**

Jacinta L. Simmons[1], Carly J. Pierce[1], Fares Al-Ejeh[2] and Glen M. Boyle[1*].

[1]Cancer Drug Mechanisms Group, Department of Cell and Molecular Biology, QIMR Berghofer Medical Research Institute, Herston, Queensland, 4006, Australia

[2]Personalised Medicine Team, Department of Cell and Molecular Biology, QIMR Berghofer Medical Research Institute, Herston, Queensland, 4006, Australia.

*** Corresponding author -** Glen M. Boyle, QIMR Berghofer Medical Research Institute, Locked Bag 2000, Post Office Royal Brisbane Hospital, QLD 4029, Australia. Tel.: +61 7 3362 0319; Fax: +61 7 3845 3508; E-mail: Glen.Boyle@qimrberghofer.edu.au

## SUPPLEMENTARY FIGURE LEGENDS

**Supplementary Figure S1. Additional data relating to Figure 1.** (**a**) Long exposure of data from Figure 1a showing MITF expression in MITF$^{low}$ cell lines C32, HT144 and MM455. (**b**) Western blot analysis of known MITF target gene *SNAI1* (encoding SNAIL) in the MITF$^{low}$ cell line MM455 shows knockdown 48 and 96 h after induction of shRNA expression.

**Supplementary Figure S2. qPCR validation of targets following knockdown of BRN2 (shBRN2) or MITF (shMITF).** Down- or up-regulated targets following induction of either shMITF or shBRN2 were identified by firstly comparing to the same vehicle treated, uninduced cells, then by comparing to shNEG versus vehicle treated uninduced cells to

account for changes potentially caused by shRNA expression or doxycycline exposure. All values are normalized to vehicle treated, uninduced shRNA cells. (**a**) Genes identified as being down-regulated with either MITF or BRN2 knockdown were validated using qPCR. (**b**) Genes identified as being up-regulated with MITF knockdown. Values indicate mean +/- SD. SD, standard deviation.

**Supplementary Figure S3. Impact of MITF or BRN2 knockdown on cellular proliferation.** (**a**) A02 or (**b**) MM455 cells were seeded into 96-well plates and induced with doxycycline for 7 days and a Sulforhodamine B assay was used to measure cellular proliferation rate. (**c**) Western blot analysis of MITF and BRN2 level in MM649 or HT144 cells after treatment with siRNA for 48 h. (**d** and **e**) Relative growth of (**d**) MM649 or (**e**) HT144 after treatment with siRNA targeting either BRN2 or MITF at 3 or 5 days post transfection. Values are normalized to control siRNA. Values indicate mean +/- SD. * $P < 0.05$, ** $P < 0.01$, **** $P < 0.0001$, unpaired t-test. SD, standard deviation.

**Supplementary Figure S4. Cell cycle profiles of MITF or BRN2 depleted cells.** Cell cycle profiles were determined by PI staining and FACS analysis 6 days after indicated shRNA expression in (**a**) HT144 and (**b**) MM649 cells. Values indicate mean +/- SD from 4 independent experiments. SD, standard deviation.

**Supplementary Figure S5. Absence of PARP-1 cleavage on MITF or BRN2 knockdown.** Western blot analysis for PARP-1 cleavage was performed on MM649 or HT144 cells, 96 h after induction of shRNA expression. BRN2 and MITF knockdown was also assessed, with GAPDH as a loading control. Doxorubicin (DOXO; HT144, 1 µg/ml; MM649, 3 µg/ml) was used to induce PARP-1 cleavage to act as a positive control. n.d., not determined.

**Supplementary Figure S6. Individual xenograft tumor volumes after induction of shRNA by doxycycline.** Raw data relating to Figure 3d and e. (**a**) MM649, (**b**) HT144. Cells with inducible depletion of MITF or BRN2 were injected sub-cutaneously into the right and left flanks of 5-week-old male BALB/c *Foxn1*$^{nu}$ (nude) mice, one shRNA per mouse. Expression of shRNA in established tumors was induced by the addition of doxycycline to drinking water when tumors reached approximately 50 mm$^3$. Data shows 10 tumors in total for MM649; 12 tumors in total for HT144. Number of tumors with zero volume are indicated with text. Black lines / text – Vehicle treatment; red lines / text – doxycycline treatment.

**Supplementary Figure S7. Immunohistochemical detection of BRN2 and MITF in primary xenograft tumors.** Haematoxylin and eosin (left panels), BRN2 (middle panels) and MITF (right panels) staining of xenograft tumors extracted, formalin fixed and paraffin embedded on day 14 post-doxycycline administration. (**a**) MM649, (**b**) HT144. Scale bars 200 µm.

**Supplementary Figure S8. BRN2 and MITF expression is required for growth after metastatic dissemination.** HT144 tumor area per lung section was calculated after complete sectioning using Genie software analysis. Data shows a significantly decreased tumor burden in both (**a**) raw area and (**b**) percentage area in mice injected with cells with reduced MITF and BRN2. Black circles, vehicle only; white squares, doxycycline. Values indicate mean +/- SEM, n = 5 mice per group, 8 sections per lung mounts. *** $P < 0.001$, **** $P < 0.0001$, Mann-Whitney test. SEM, standard error of the mean.

**Supplementary Figure S9. Full-length blots from Figure 1a, c.**

**Supplementary Figure S10. Full-length blots from Figure 1d-f.**

**Supplementary Figure S11. Full-length blots from Figure 3f.**

## SUPPLEMENTARY TABLES

**Supplementary Table S1. Differential gene expression following induction of shMITF with doxycycline for 96 hours.** All 4 melanoma cell lines are shown in individual sheets. Data was generated by initially comparing to the cells treated with vehicle alone, and then fold change relative to cells expressing shNEG, again doxycycline versus vehicle.

**Supplementary Table S2. Differential gene expression following induction of shBRN2 with doxycycline for 96 hours.** All 4 melanoma cell lines are shown in individual sheets.

**Supplementary Table S3. Upstream regulator pathway analysis from IPA following induction of shMITF with doxycycline for 96 hours.** All 4 melanoma cell lines are shown in individual sheets.

**Supplementary Table S4. Functional annotation pathway analysis from IPA following induction of shMITF with doxycycline for 96 hours.** All 4 melanoma cell lines are shown in individual sheets.

**Supplementary Table S5. Upstream regulator pathway analysis from IPA following induction of shBRN2 with doxycycline for 96 hours.** All 4 melanoma cell lines are shown in individual sheets.

**Supplementary Table S6. Functional annotation pathway analysis from IPA following induction of shBRN2 with doxycycline for 96 hours.** All 4 melanoma cell lines are shown in individual sheets.

**Supplementary Table S7. Selected Upstream Regulator pathways showing data from both shMITF and shBRN2 induction in all 4 melanoma cell lines.** Average z-score and *P* value are also shown.

**Supplementary Table S8. Selected Functional Annotation pathways showing data from both shMITF and shBRN2 induction in all 4 melanoma cell lines.** Average z-score and *P* value are also shown.

**Supplementary Table S9. Primer sequences used in this study.**

| Target | Forward (5' to 3') | Reverse (5' to 3') |
|---|---|---|
| *MITF* | TGCTGGAAATGCTAGAATATAATCACT | ATGACATGATCGCCAGGCTG |
| *MAP3K11* | TCCCCCTTAGGATCTCCTTC, | GACAGGCCTCTTGGGCTC |
| *CEACAM1* | GGGACGTATTGGTGTGAGGT | GAGAGGCCATTTTCTTGTGG |
| *SPARC* | CTTCAGACTGCCCGGAGA | GAAAGAAGATCCAGGCCCTC |
| *SKP2* | GCTGAAGAGCAAAGGGAGTG | GAAGGGAGTCCCATGAAACA |
| *RTN4* | CGTGACAAGAGATGGACGGT | AATAGGCTGGCACCAAACAC |
| *SNAI2* | CAGACCCTGGTTGCTTCAA | TGACCTGTCTGCAAATGCTC |
| *NFIC* | TGGACCTCTACCTGGCCTAC | CTTGCTGTCCTCCTGGTCA |
| *AXL* | ACCTACTCTGGCTCCAGGATG | CGCAGGAGAAAGAGGATGTC |
| *IL1A* | ACTGCCCAAGATGAAGACCA | CCGTGAGTTTCCCAGAAGAA |
| *IL6* | AGTGAGGAACAAGCCAGAGC | GTCAGGGGTGGTTATTGCAT |
| *IL8* | TCCTGATTTCTGCAGCTCTGT | AAATTTGGGGTGGAAAGGTT |
| *GAPDH* | GGCTCTCCAGAACATCATCCCTGC | GGGTGTCGCTGTTGAAGTCAGAGG |

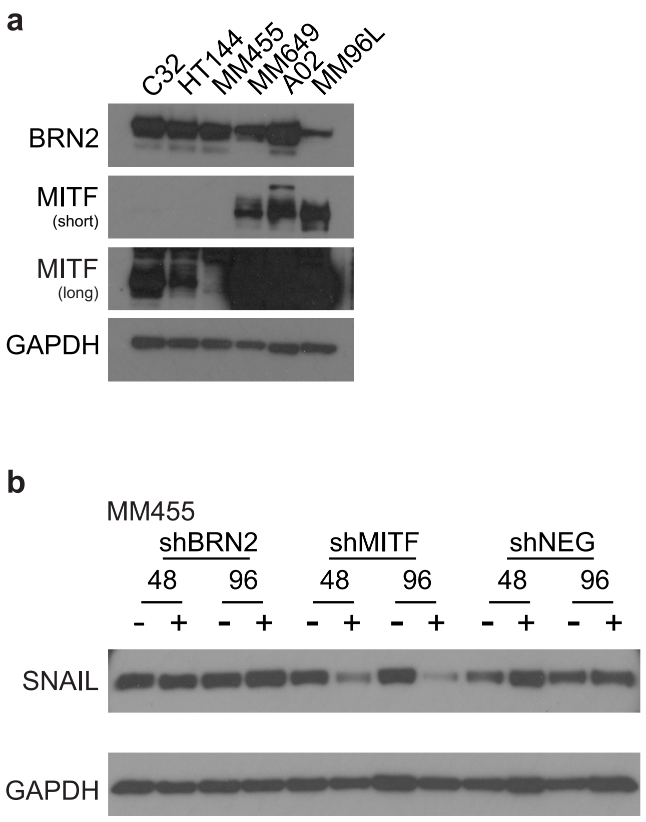


**Supplementary Figure S1**

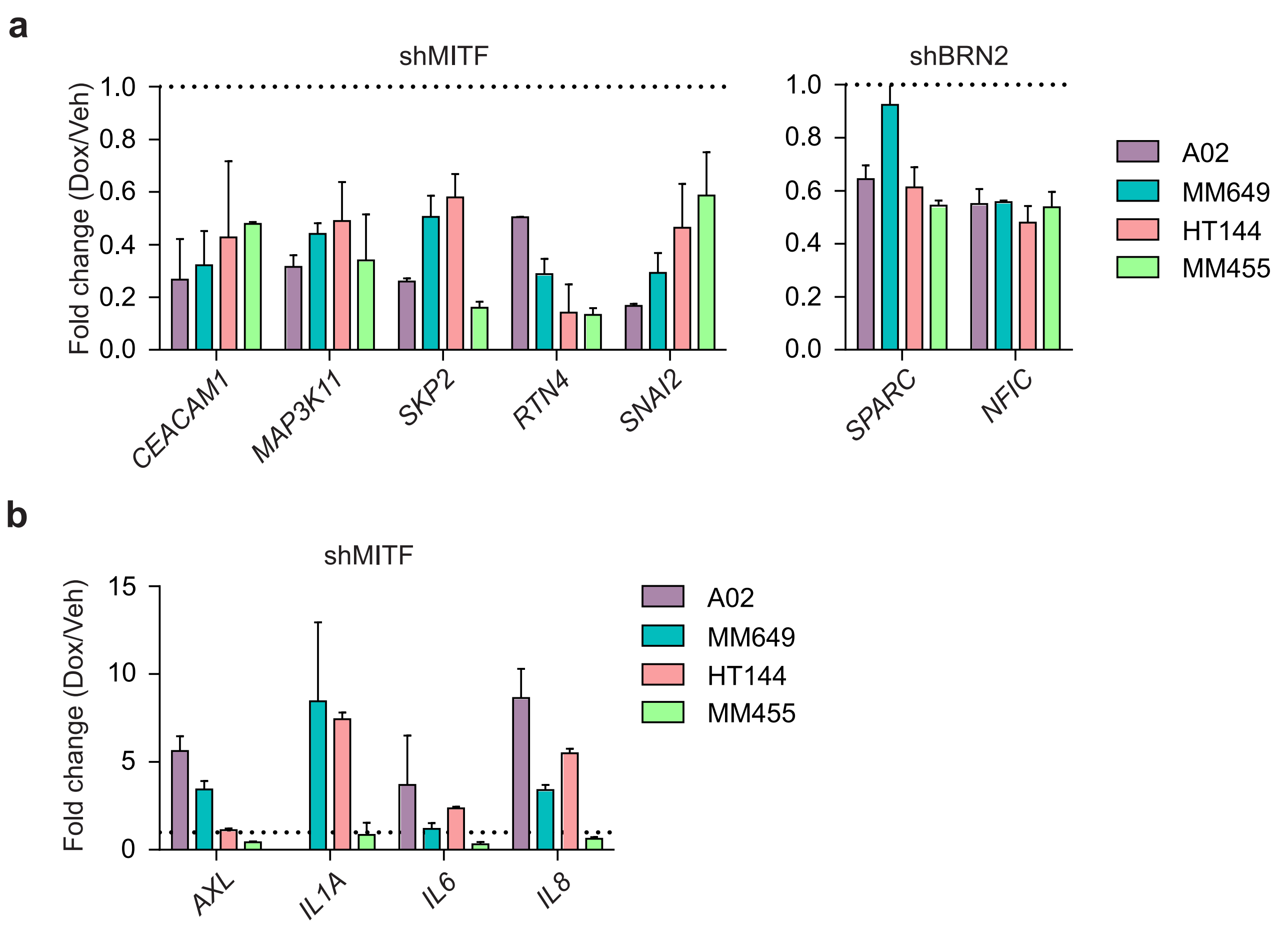


**Supplementary Figure S2**

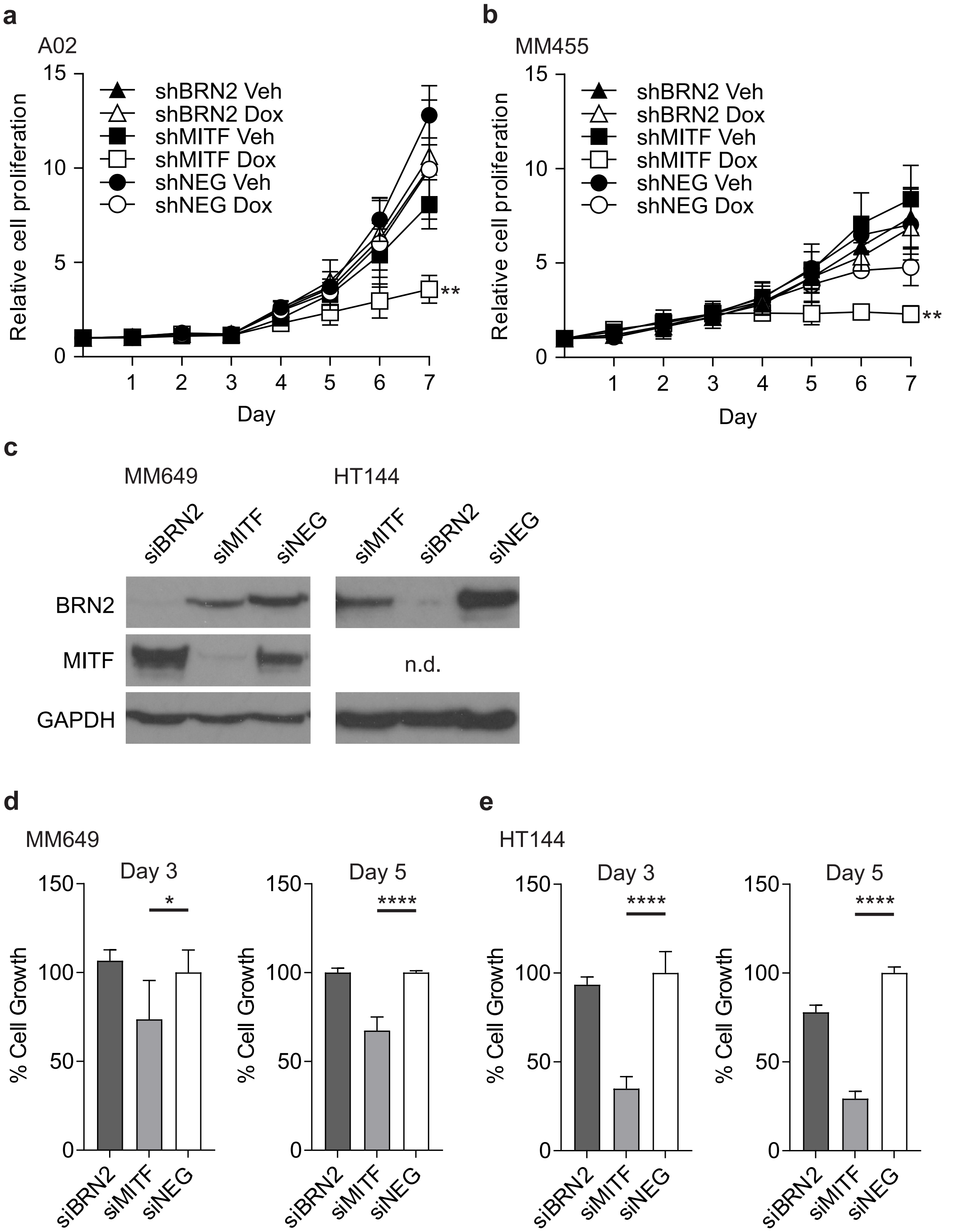


**Supplementary Figure S3**

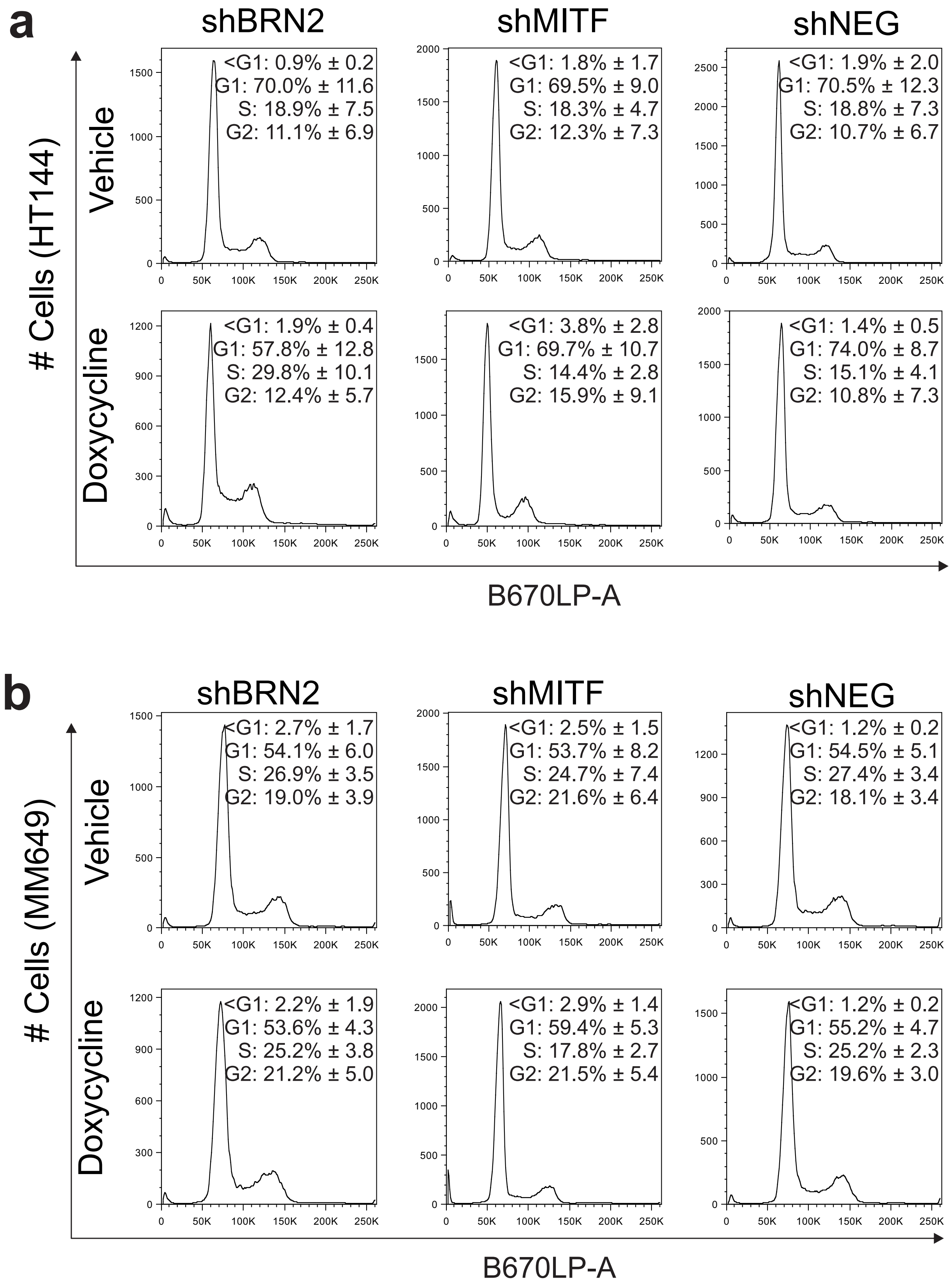


**Supplementary Figure S4**

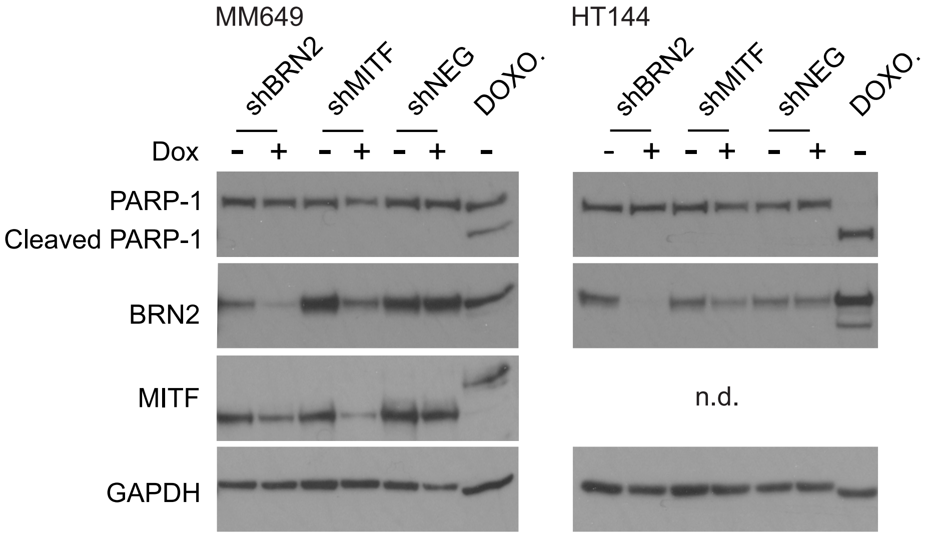


**Supplementary Figure S5**

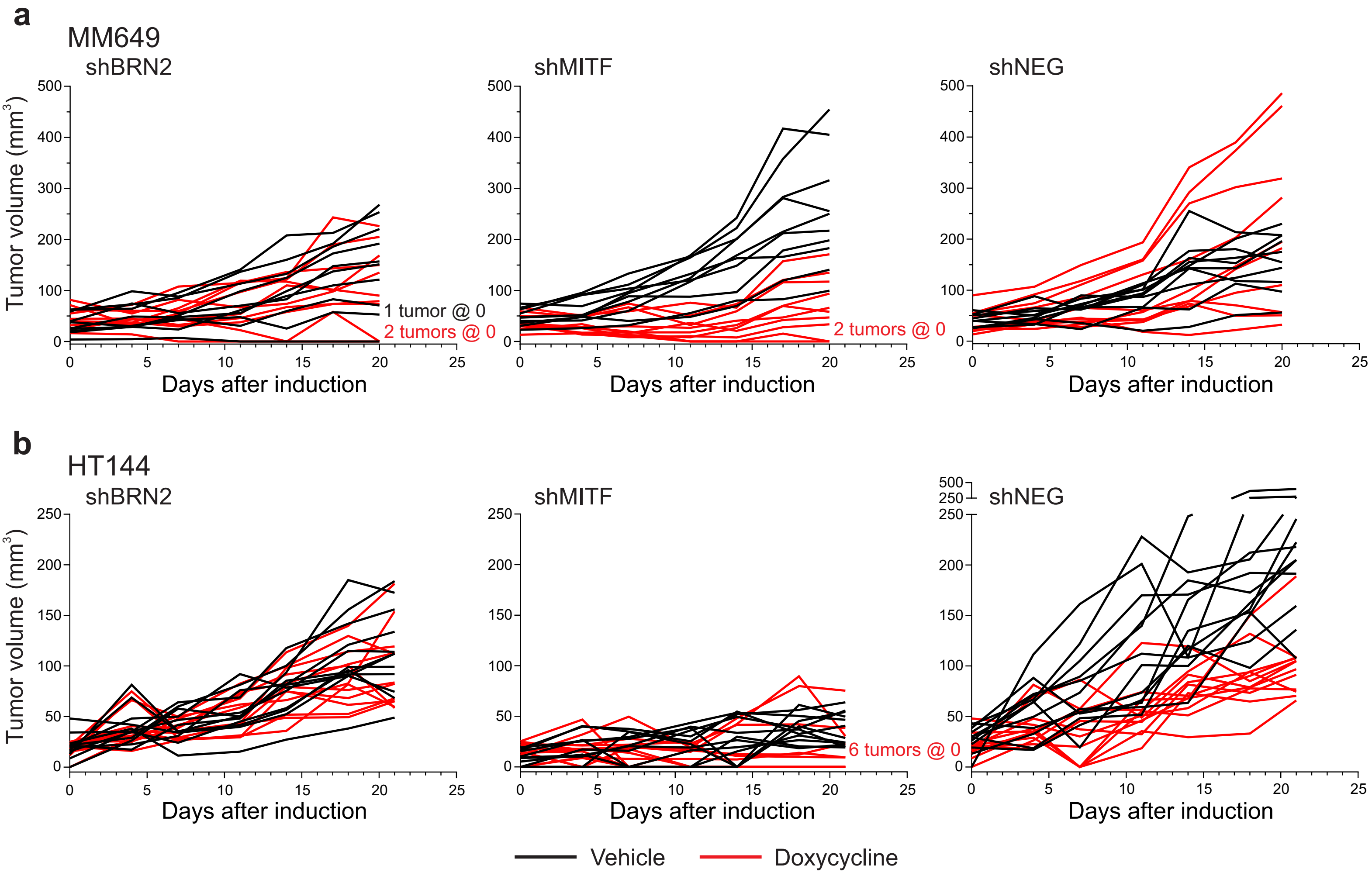


**Supplementary Figure S6**

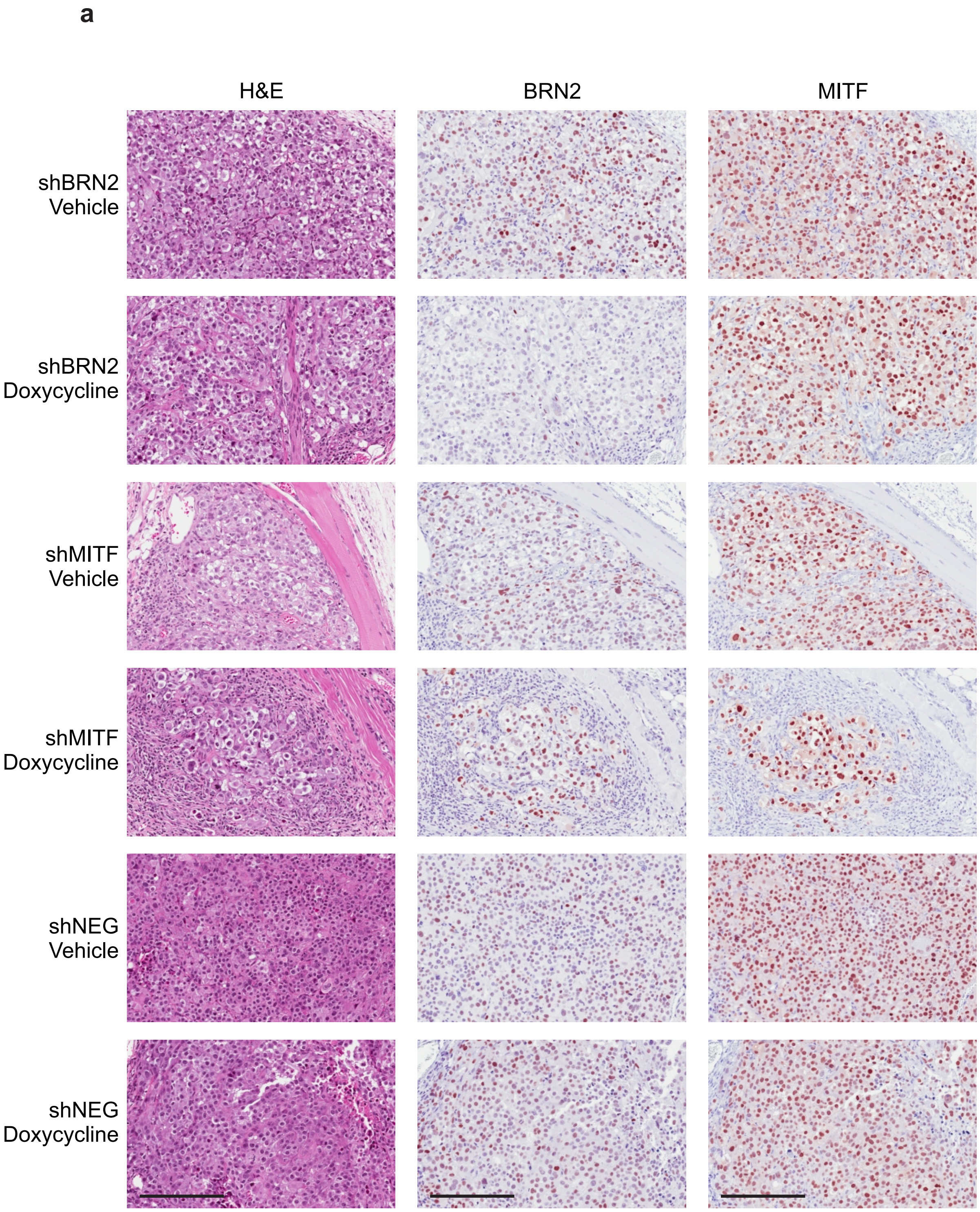


**Supplementary Figure S7**

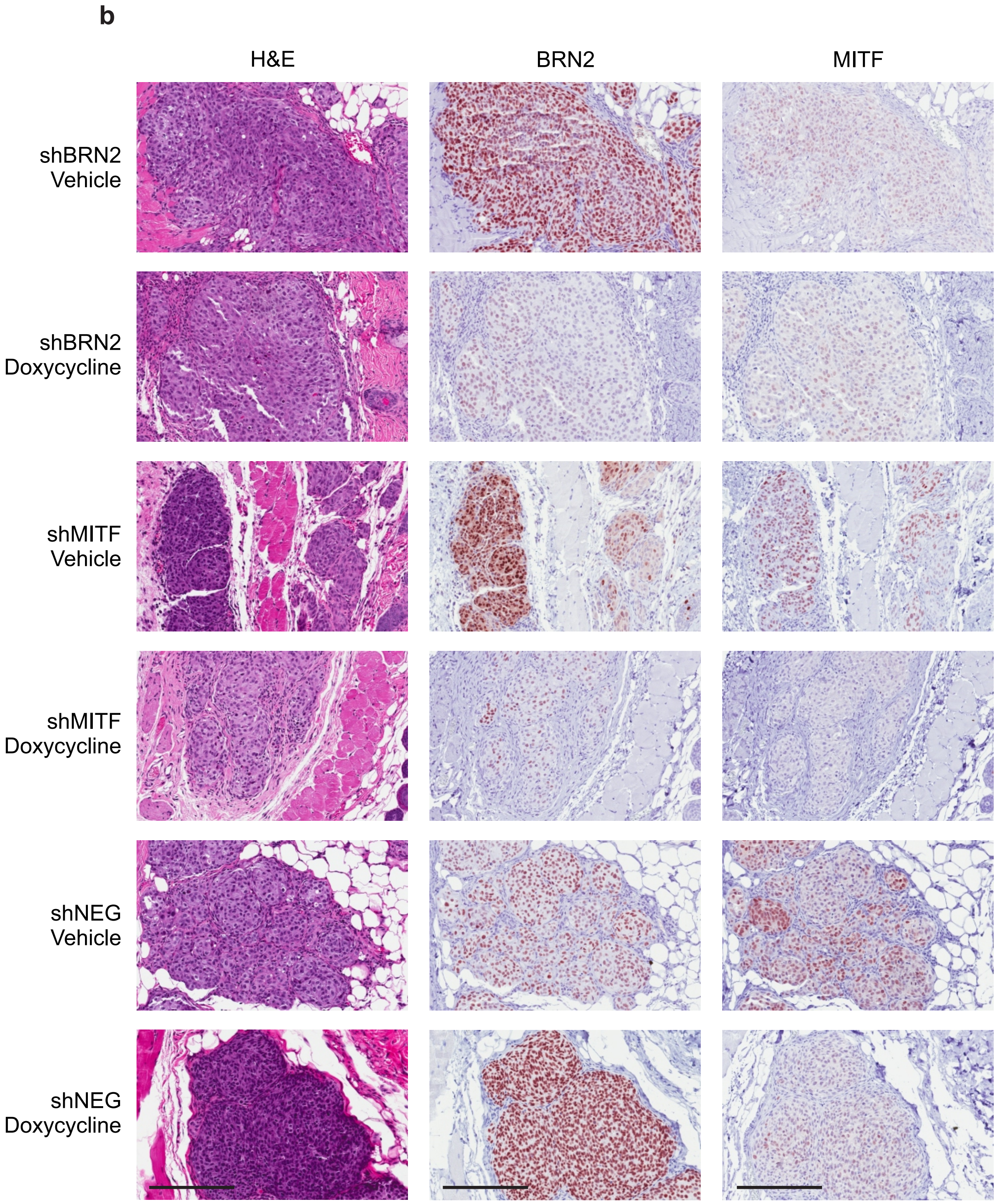


**Supplementary Figure S7**

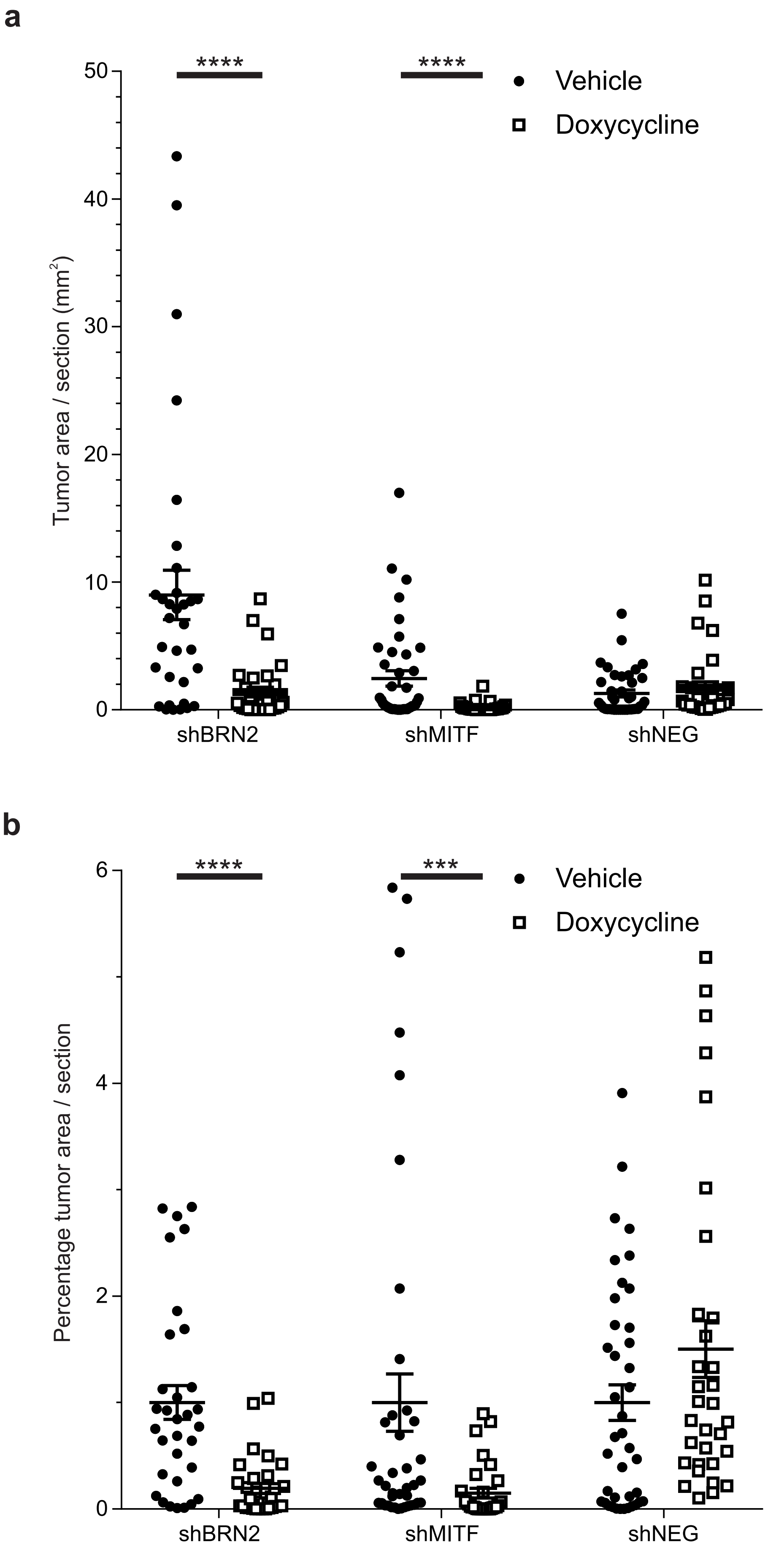


**Supplementary Figure S8**

## Full length blots for Figure 1a

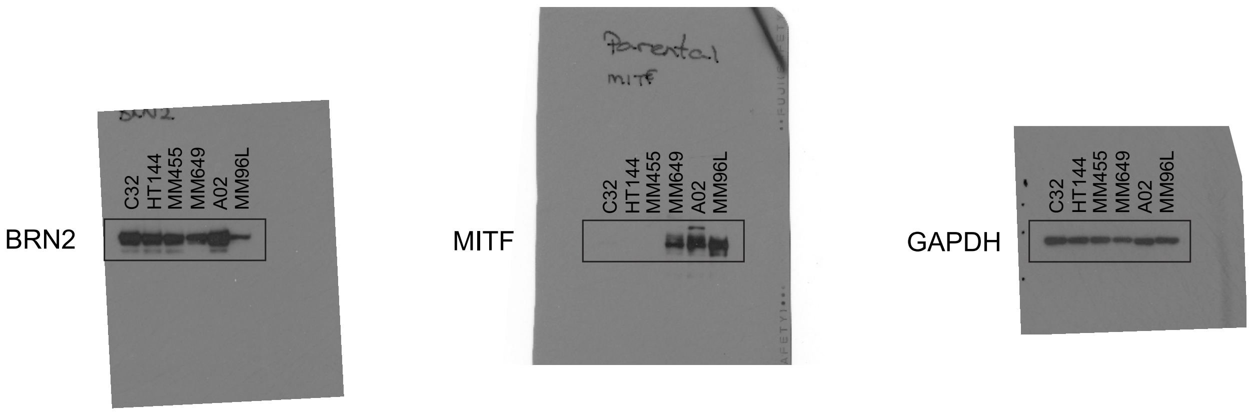


## Full length blots for Figure 1c

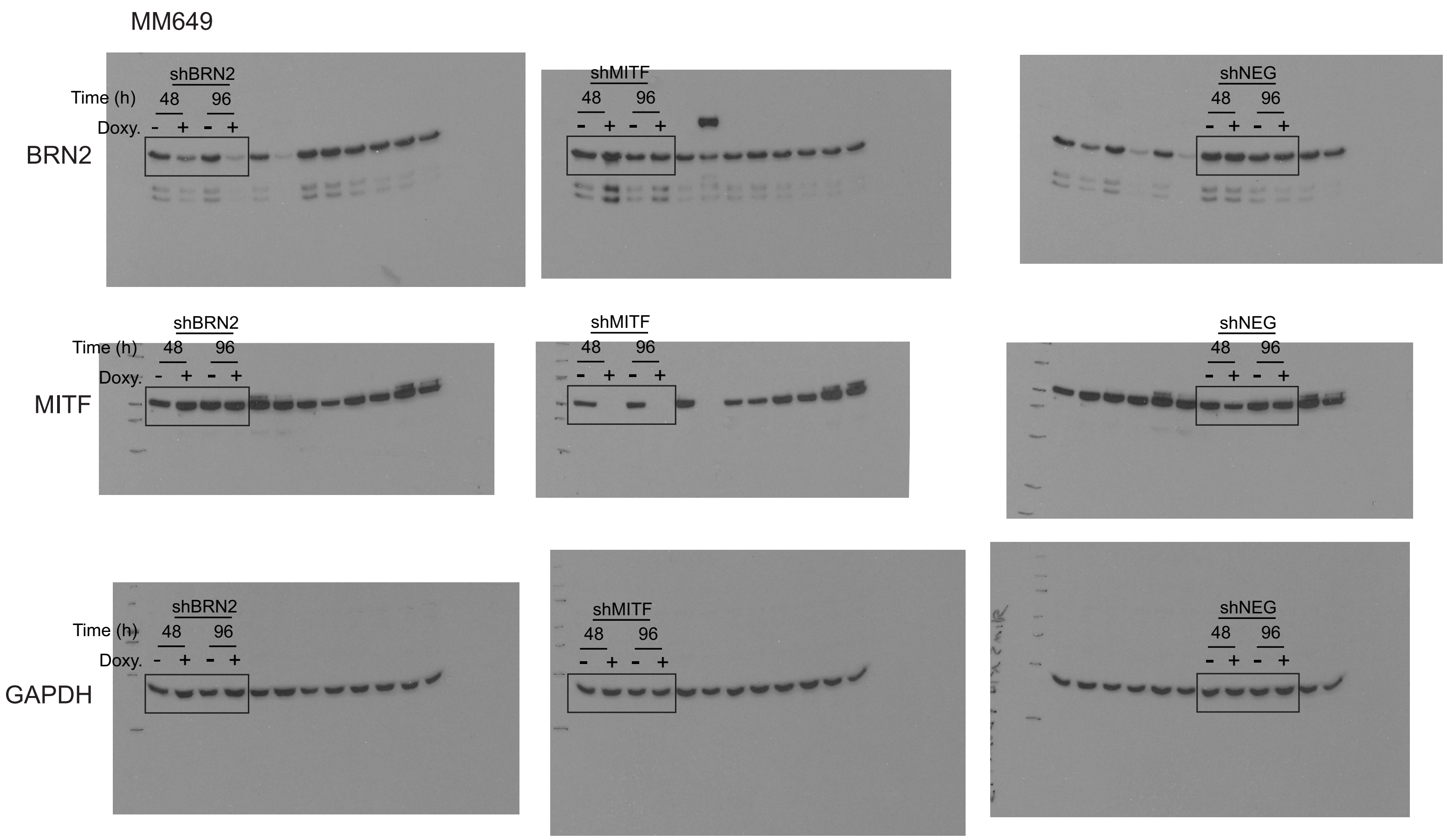


**Supplementary Figure S9.** Full-length blots from Figures 1a, c.

## Full length blots for Figure 1d

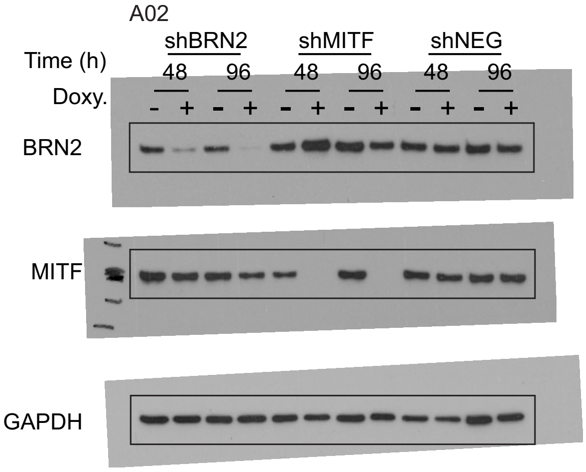


## Full length blots for Figure 1e

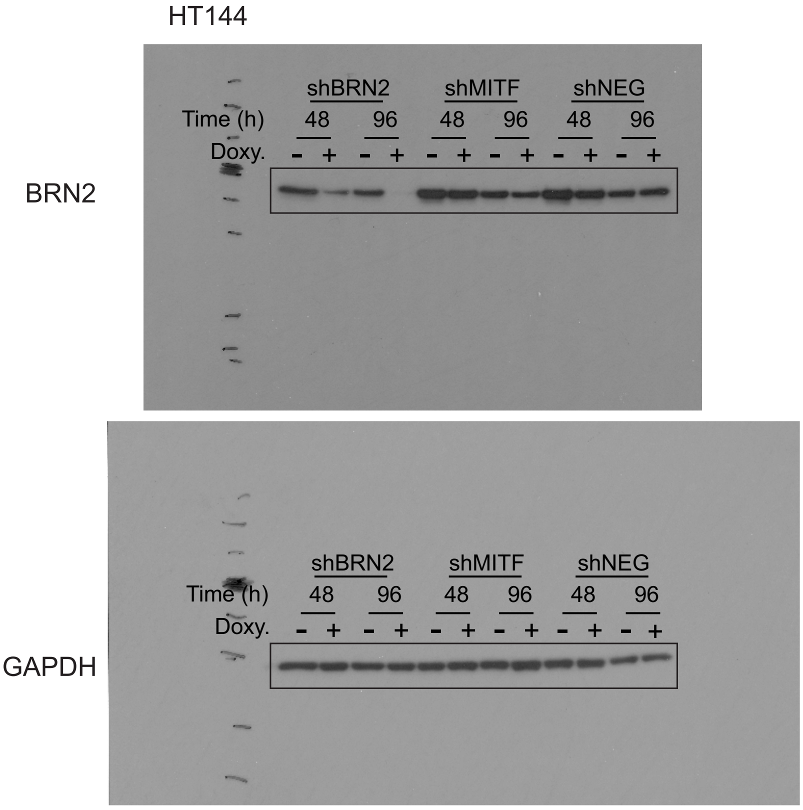


## Full length blots for Figure 1f

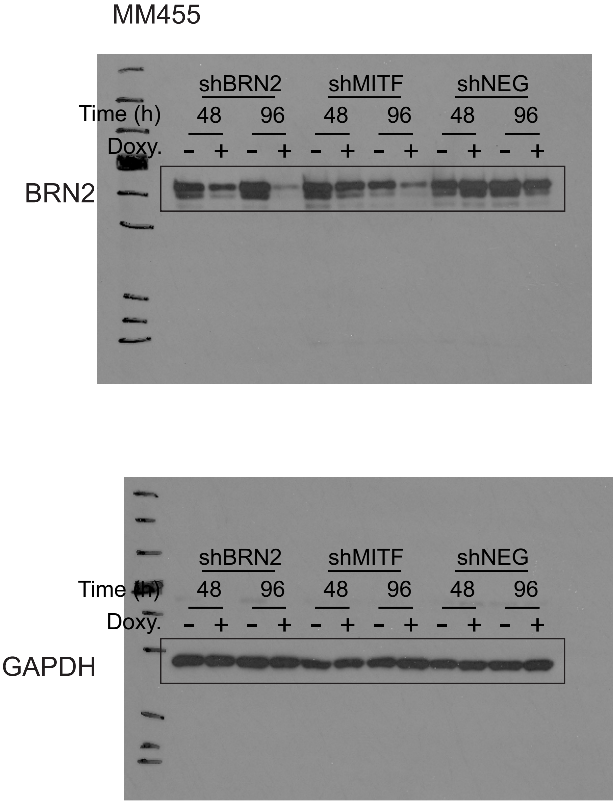


**Supplementary Figure S10.** Full-length blots from Figures 1d-f.

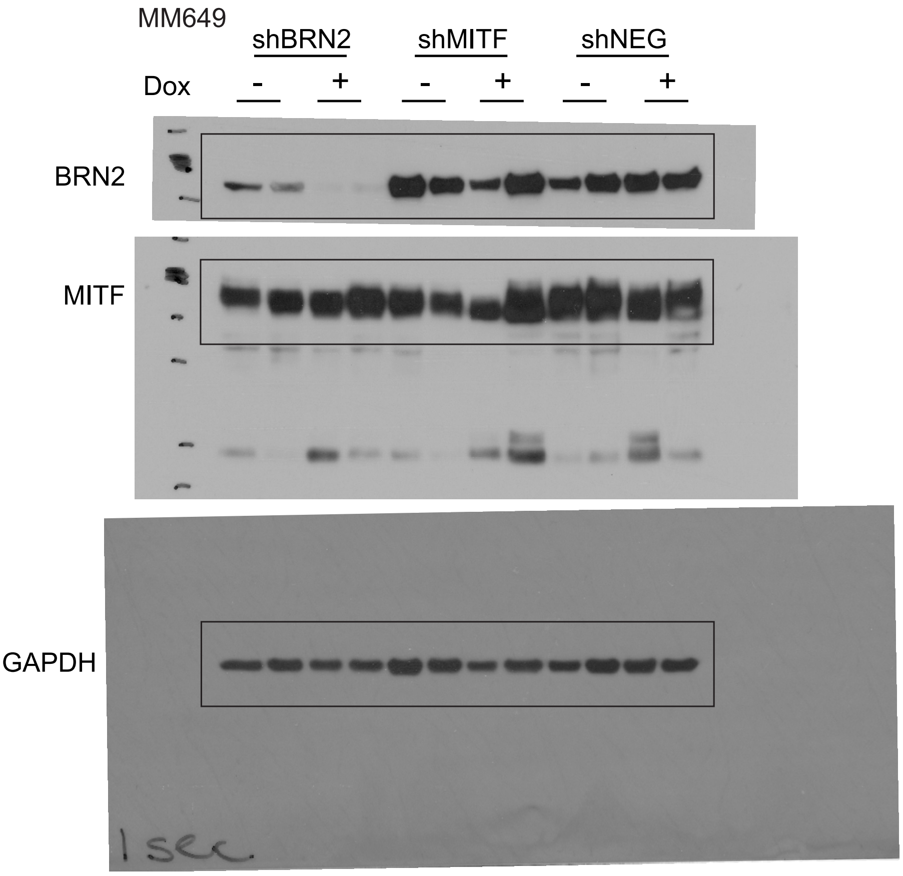


**Supplementary Figure S11.** Full-length blots from Figure 3f.
